# Effect of Cryo-Processing on Platelet-Rich Autoplasma Preparations

**DOI:** 10.17691/stm2020.12.6.07

**Published:** 2020-12-28

**Authors:** D.V. Melnikov, K.A. Kirillova, A.S. Zakharenko, M.Y. Sinelnikov, A.A. Ragimov, A.L. Istranov, O.I. Startseva

**Affiliations:** Associate Professor, Department of Oncology, Radiotherapy and Plastic Surgery; I.M. Sechenov First Moscow State Medical University (Sechenov University), 8/2 Trubetskaya St., Moscow, 119991, Russia; Junior Researcher, Department of Oncology, Radiotherapy and Plastic Surgery; I.M. Sechenov First Moscow State Medical University (Sechenov University), 8/2 Trubetskaya St., Moscow, 119991, Russia; Junior Researcher, Department of Oncology, Radiotherapy and Plastic Surgery; I.M. Sechenov First Moscow State Medical University (Sechenov University), 8/2 Trubetskaya St., Moscow, 119991, Russia; Junior Researcher, Institute of Regenerative Medicine; I.M. Sechenov First Moscow State Medical University (Sechenov University), 8/2 Trubetskaya St., Moscow, 119991, Russia; Professor, Department of Clinical Transfusion Medicine; I.M. Sechenov First Moscow State Medical University (Sechenov University), 8/2 Trubetskaya St., Moscow, 119991, Russia; Professor, Department of Oncology, Radiotherapy and Plastic Surgery; I.M. Sechenov First Moscow State Medical University (Sechenov University), 8/2 Trubetskaya St., Moscow, 119991, Russia; Professor, Department of Oncology, Radiotherapy and Plastic Surgery I.M. Sechenov First Moscow State Medical University (Sechenov University), 8/2 Trubetskaya St., Moscow, 119991, Russia

**Keywords:** cell technologies, platelet growth factor, PDGF, platelet alpha granules, proliferation, activated P-PRP, L-PRP, non-activated P-PRP, L-PRP

## Abstract

Platelet-derived growth factor (PDGF) plays an important role in angiogenesis, affects activation of migration and proliferation of mesenchymal stem cells, fibroblasts, smooth muscle cells, osteoblasts; activation of migration of monocytes, macrophages, and neutrophils.

**The aim of the investigation** was to study the effect of cryo-processing on the qualitative properties of platelet-rich autoplasma (PRP) at different time intervals.

**Materials and Methods.:**

Autologous plasma preparations were obtained from the blood of 31 donors. The biological material was prepared by double centrifugation according to the protocol for obtaining P-PRP and L-PRP. Platelet count and the concentration of growth factors (PDGF-AA and PDGF-BB) were studied in fresh PRP preparations. In frozen PRP samples, the concentration of PDGF-AA and PDGF-BB was determined 2 weeks after cryo-processing and 2 months after cryo-processing at –35 °С. P-PRP and L-PRP samples activated with 10% CaCl^2^ solution and those non-activated were studied.

**Results.:**

L-PRP preparations are significantly superior to P-PRP preparations: the concentration of platelets is 1.7 times higher in them. The level of PDGF-AA in non-activated L-PRP is 1.8 times higher than in non-activated P-PRP (p<0.05). The level of PDGF-AA is 1.5 times higher in activated L-PRP than in activated P-PRP (p<0.05). The level of PDGF-BB is 2.9 times higher in non-activated L-PRP than in non-activated P-PRP and 1.8 times higher in activated L-PRP than in activated P-PRP (p<0.05). The concentration of PDGF-BB in non-activated P-PRP sharply increases in the 2^nd^ week after freezing and remains at the same level after 2 months (p<0.05). The concentration of PDGF-BB in activated plasma does not change (p>0.05).

**Conclusion.:**

Cryo-processing of non-activated autologous L-PRP allows preserving and subsequently enhancing the properties of plasma concentrate, which makes it possible to apply it in clinical practice.

## Introduction

One of the most promising areas in plastic surgery is transplantation of autologous adipose tissue to replenish lost tissue volumes, to carry out aesthetic correction, or to repair and regenerate body tissues [1–3]. Despite the ease and safety, this method has a significant drawback of unpredictable and ineffective engraftment of the transplanted fat [4].

Advances in cell technology provide the possibility to look at the problem of transplanting autologous adipose tissue from a different angle. Particular attention is paid to the factors influencing survival rates of fat autografts. To improve the survival rate of freely transplanted fat, preparations of autologous platelet-rich plasma (PRP) are used [5–7].

Application of blood components for non-transfusion purposes was started after the report by Marx et al. [5, 8] on the successful use of thromboconcentrate in dentistry. According to Marx, PRP is blood plasma with platelet concentration significantly higher than normal [9]. Normally, it is in the range of 150–350 thousand cells/μl and, on average, it is 200 thousand cells/μl. It has been proven that the stimulating effect of platelet-rich autoplasma becomes obvious, if its platelet concentration equals 1 million cells/μl or more [10]. In a number of studies, PRP platelet concentration was increased 2–8.5 times the norm [5, 7, 11–15].

As an autologous product, platelet-rich plasma is advantageous for therapeutic use as it poses no risk of side effects and no risk of transmitting infectious diseases [5, 6, 16, 17].

Platelet-rich plasma uniqueness and specificity are attributed to the multifactorial local influence of a highly concentrated complex of biological mediators [10]. PRP growth factors are released from platelet alpha granules in response to their activation [15, 18, 19]. The main growth factors are platelet growth factor (PDGF-AA, PDGF-BB, PDGF-AB), transforming growth factor (TGF-β1 and TGF-β2), vascular endothelial growth factor (VEGF), fibroblast growth factor (FGF), insulin-like growth factor (IGF-1 and IGF-2), and epithelial growth factor (EGF) [10, 11].

Platelet growth factor plays an important role in angiogenesis. PDGF receptors are located in the vascular wall on fibroblasts and smooth muscle cells. PDGF stimulates proliferation of these cells. This factor is involved in activation of migration and proliferation of mesenchymal stem cells, fibroblasts, smooth muscle cells, osteoblasts; activation of migration of monocytes, macrophages, neutrophils [10, 14].

The efficacy of using this substance (in particular, its beneficial effect on target tissues) is associated with platelets. Platelet alpha granules are known to contain growth factors affecting target tissues; therefore, the number of growth factors depends directly on the number of platelets. The cellular composition of platelet-rich plasma has a significant effect on the concentration of platelets and, accordingly, growth factors [20].

Platelet-rich autoplasma preparations are divided into four categories based on leukocyte and fibrin content: pure platelet-rich autoplasma (P-PRP); leukocyte- and platelet-rich autoplasma (L-PRP); pure platelet-rich fibrin (P-PRF); leukocyte- and platelet-rich fibrin (L-PRF) [21–23].

Storage (freezing) of platelet-rich autoplasma is a popular subject of debates and numerous publications. Freezing and thawing do not require blood sampling for each injection in case of several treatment sessions, but they impair platelet function, thereby altering the pattern of growth factor release [6]. For these reasons, fresh PRP administration is considered more effective [24].

**The aim of the investigation** was to study the effect of cryo-processing on the qualitative properties of platelet-rich autoplasma at different time intervals.

## Materials and Methods

The work was carried out at I.M. Sechenov First Moscow State Medical University (Russia). Autologous plasma preparations (P-PRP and L-PRP) were obtained from the blood of 31 volunteer donors (mean age 38.0± 13.2 years).

Two 9 ml vacutainer tubes of blood with 3.8% concentration of Na_3_C_6_H_5_O_7_ were drawn from donors. The tubes were marked with an individual barcode assigned to each donor on registration. Clinical analysis was performed to determine the baseline parameters of the cellular composition of blood. Next, the tubes with the biological material were centrifuged twice according to the protocol for obtaining P-PRP and L-PRP (500 g for 5 min and 1538 g for 3 min, where g is centrifugal acceleration value). The protocol proven by us earlier [25] allows achieving platelet concentration of 1,900,000 to 2,436,000 per 1 μl and leukocyte concentration (as part of L-PRP) of 10,000 to 15,000 per 1 μl, which is a key aspect for the effective use of these preparations.

Platelet count and the concentration of growth factors (PDGF-AA and PDGF-BB) were studied in fresh PRP preparations. In frozen PRP samples (P-PRP and L-PRP), the concentration of PDGF-AA and PDGF-BB was determined 2 weeks after cryo-processing and 2 months after cryo-processing. The temperature regime was chosen with regard to the rules for cryo-processing of protein fractions (from –20 to –80°C). The value of –35°С was chosen as the average, taking into account the characteristics of the laboratory equipment. Eppendorf laboratory tubes were used for sampling. All the samples obtained were investigated in two states: samples activated with 10% CaCl_2_ solution in the ratio of 1:10 and those non-activated.

The composition of autoplasma (the presence and count of leukocytes and platelets) was determined using the Sysmex XT2000i hematology analyzer (Sysmex, Japan). The concentration of growth factors (FGF basic, PDGF-AA, PDGF-BB, VEGF) was studied using the xMAP Luminex technology (Gen-Probe, USA) based on flow cytometry (fluorimetry). This method involves multiplex analysis, in which the carrier is polystyrene microspheres integrated by two fluorophores at different concentrations. A specific ratio of their concentrations creates a combination of possible types of particles with their particular unique spectral characteristic allowing simultaneous detection of several analytes in one sample. The study was carried out using the Human Angiogenesis Base Kit A test systems (R&D Systems, USA), microspheres Luminex Performance assay FGF basic, PDGF-AA, PDGF-BB, VEGF (R&D Systems) in accordance with the manufacturer’s protocol with due account for its adaptation for the use of citrated plasma (units of growth factor concentration measurement are grams per milliliter).

**Statistical data processing.** Data on blood concentration of platelets and growth factors (PDGF-AA and PDGF-BB), P-PRP and L-PRP were statistically analyzed using Statistica 8.0 (StatSoft) and SigmaStat 3.5 (Systat Software). Normality of distribution was assessed using the Kolmogorov–Smirnov test. Intergroup comparisons at a critical level of significance p<0.05 were carried out using parametric tests (Student’s t-test for paired comparisons, one-way ANOVA and Holm–Sidak t-test for multiple comparisons) in the case of normal distribution; data were presented as mean ± standard deviation of the mean. In the case of non-normal data distribution, nonparametric tests were used (Mann–Whitney U test for paired comparisons, Kruskal–Wallis H test and Dunn’s test for multiple comparisons); data were presented as Me (min–max), where Me is the median; min, max are the minimum and maximum values.

## Results

**Figure 1:**
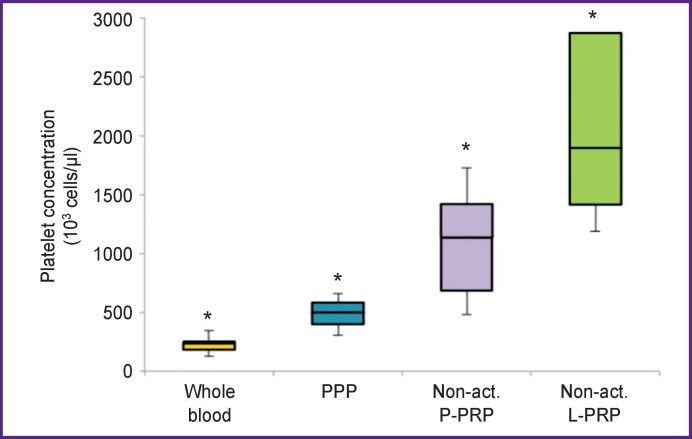
Platelet concentration in peripheral blood, PPP, non-activated P-PRP and L-PRP * Intergroup differences in values are statistically significant, p<0.05

***Platelet concentration*** in whole blood was 238 (183–254)·10^3^ cells/μl; it was 500 (400–584)·10^3^ cells/ μl in platelet-poor plasma (PPP) after the 1^st^ stage of centrifugation; in P-PRP — 1138 (690–1422)·10^3^ cells/ μl; in L-PRP — 1900 (1418–2875)·10^3^ cells/μl. L-PRP showed an increase in platelet count by 1.7 times as compared with P-PRP (p<0.05) (Figure 1).

### The concentration of growth factors.

**Figure 2:**
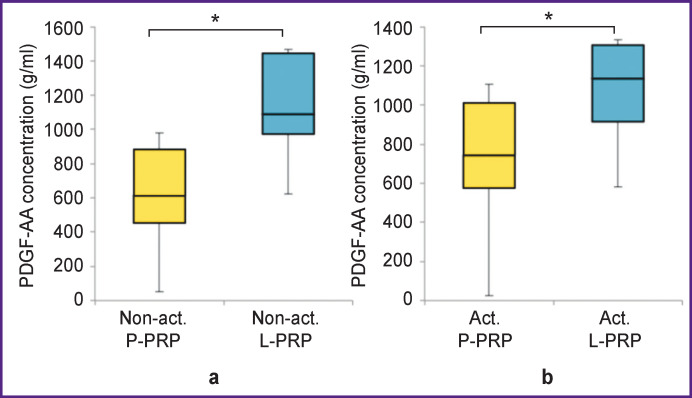
Concentration of PDGF-AA growth factor in non-activated (a) and activated (b) P-PRP and L-PRP; * p<0.05

The concentration of PDGF-AA in non-activated L-PRP (1089.9 (973.1–1444.1) g/ml) was 1.8 times higher than in non-activated P-PRP (614.7 (453.6–885.8) g/ml) (p<0.05). The concentration of PDGF-AA in activated L-PRP (1134.3 (914.9–1305.6) g/ml) was 1.5 times higher than in activated P-PRP (742.5 (575.6–1008.8) g/ml) (p<0.05) (Figure 2).

**Figure 3:**
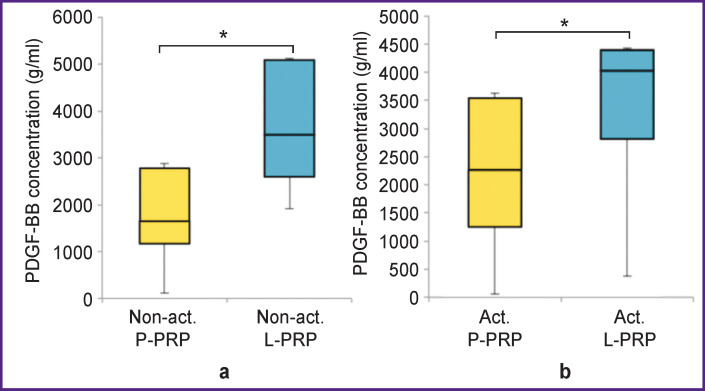
Concentration of PDGF-BB growth factor in non-activated (a) and activated (b) P-PRP and L-PRP; * p<0.05

The concentration of PDGF-BB in non-activated L-PRP (3493.2 (2594.3–5086.9) g/ml) was 2.9 times higher than in non-activated P-PRP (1651.7 (1168.2–2785.2) g/ml) (p<0.05). The concentration of PDGF-BB in activated L-PRP (4029.1 (2814.1–4393.1) g/ml) was 1.8 times higher than in activated P-PRP (2262.1 (1250.0–3539.6) g/ml) (p<0.05) (Figure 3).

### Changes in growth factors after freezing.

**Figure 4:**
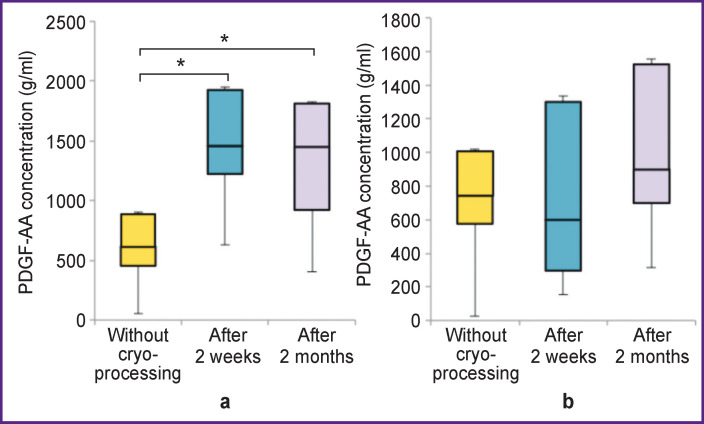
Correlation between PDGF-AA growth factor concentration and the freezing period: (a) non-activated P-PRP, * p<0.05; (b) activated P-PRP

The concentration of PDGF-AA in non-activated P-PRP increased 2 weeks after freezing and remained at the same level after 2 months (p<0.05). There were no significant changes in the concentration of PDGF-AA in activated P-PRP (p>0.05) (Figure 4).

**Figure 5:**
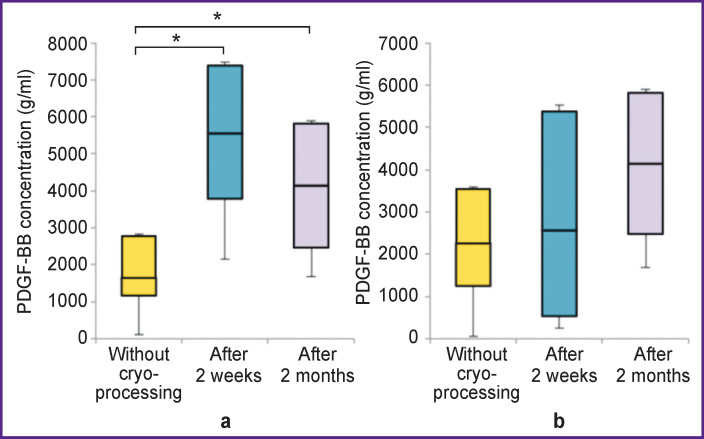
Correlation between PDGF-BB growth factor concentration and the freezing period: (a) non-activated P-PRP, * p<0.05; (b) activated P-PRP

The concentration of PDGF-BB in non-activated P-PRP increased sharply in week 2 after freezing and remained much higher than the baseline values after 2 months (p<0.05). There were no significant changes in the concentration of PDGF-BB in activated plasma (p>0.05) (Figure 5).

**Figure 6:**
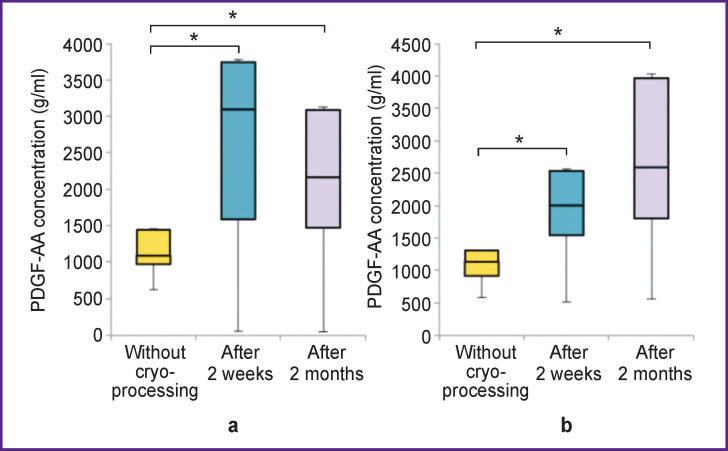
Correlation between PDGF-AA growth factor concentration and the freezing period: (a) non-activated L-PRP; (b) activated L-PRP; * p<0.05

The concentration of PDGF-AA in non-activated L-PRP increased after 2 weeks and remained above the baseline values after 2 months (p<0.05). The concentration of PDGF-AA in activated plasma increased statistically significantly after 2 weeks, remaining above the baseline level after 2 months (p<0.05) (Figure 6).

**Figure 7:**
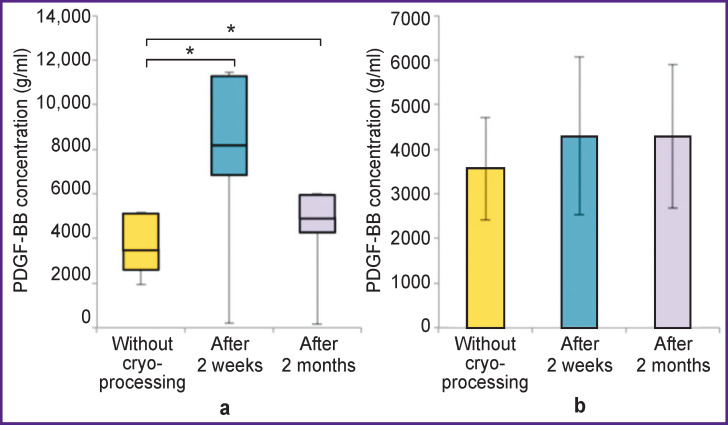
Correlation between PDGF-BB growth factor concentration and the freezing period: (a) non-activated L-PRP, * p<0.05; (b) activated L-PRP

The concentration of PDGF-BB in non-activated L-PRP increased statistically significantly 2 weeks after freezing, and decreased after 2 months, while remaining above the baseline level (p<0.05). The concentration of PDGF-BB in activated plasma remained the same at all stages of the study (p>0.05) (Figure 7).

## Discussion

Platelet-rich plasma is an autologous human blood plasma rich in platelets whose α-granules contain a huge quantity of growth factors promoting natural tissue regeneration. It has been established that pro-angiogenic factors released after platelet activation (VEGF, HGF, TGF-β1, βFGF, PDGF-A, -B, and -C, angiopoietin, etc.) induce migration and proliferation of endothelial cells, as well as vascular formation [26–28]. Tissue restoration and reparative regeneration are promoted due to broad-spectrum action of PRP growth factors [5, 13, 17].

There is an indisputable need for standardization of PRP medications from the stage of their preparation to clinical use, as well as finding possibilities of storing these medications, since some researchers avoid freezing/thawing for fear of potentially harmful effects on platelet function and growth factors [6].

The aim of our investigation was to test whether it was possible to apply PRP preparations after cryopreservation using the example of platelet growth factor (PDGF-AA and PDGF-BB).

PDGF-AA and PDGF-BB play an important role in the activation of migration and proliferation of cellular tissue components [7]. We measured levels of PDGF-AA and PDGF-BB in fresh and cryopreserved preparations (P-PRP and L-PRP) in both activated and non-activated samples. Levels of PDGF-AA and PDGF-BB were analyzed in each group to compare growth factor concentration.

The data obtained showed elevated levels of PDGF-AA and PDGF-BB factors in L-PRP compared to P-PRP both in the initial concentrate and after activation and cryopreservation. These results confirm the data on artificially induced polymerization in P-PRP, leading to the immediate release of growth factors and their utilization, which is lacking in L-PRP [29]. The mechanisms of this process are not fully understood, however, there is an assumption [30] that depletion of the characteristic structural units of whole blood results in dysregulation of the relationship between activating, inhibiting, and other regulatory factors affecting growth factor action. Therefore, the technology of preparing P-PRP implies the absence or minimal proportion of leukocyte elements, which probably continue to play a key role in regulating growth factor release outside the bloodstream. To improve the technology, further investigation of L-PRP features is necessary.

To determine growth factor activity duration in L-PRP and P-PRP fractions, cryopreservation was performed. The results showed that L-PRP retained growth factor activity (PDGF-AA) significantly longer than P-PRP, regardless of activation. However, there was an important consistency revealed in P-PRP growth factor activity. During cryopreservation, growth factor activity increased by 2 weeks, which suggests inhibition of spontaneous activation processes due to low temperatures. The same trend was observed in L-PRP preparations.

Definite preservation of cryo-processed plasma activity established by the authors is potentially associated with partial destruction of platelets after cryopreservation and additional release of growth factors. Further investigation of processes discussed in this article should involve identification of specific leukocyte populations. Moreover, it is necessary to determine the spectrum of other cells in PRP preparations, such as circulating stem cells that may play a key role in the activity of fractions. In-depth study of the cellular-protein composition of PRP fractions is likely to lead to new classifications and contribute to more adequate clinical application. Unification of processes for obtaining various PRP fractions is a necessary step for further clinical studies.

## Conclusion

L-PRP preparations are significantly superior to P-PRP preparations: firstly, the concentration of platelets is much higher in them, and secondly, the concentration of PDGF-AA and PDGF-BB growth factors is statistically significantly higher in L-PRP preparations as compared to P-PRP preparations.

Levels of PDGF-AA and PDGF-BB growth factors increase during cryo-processing of non-activated autoplasma fractions. Thus, cryo-processing of non-activated autoplasma can be applied successfully for preservation and subsequent effective use of plasma concentrate.

The results of the investigation confirm both theoretical and applied grounds for the use of L-PRP in clinical practice. Nevertheless, there are also possibilities to use PRP in other modifications. PRP preparations should be selected by a practitioner based on the goals and objectives to be achieved with this technology.

It is necessary to carry out further research in order to develop a unified method for obtaining PRP preparations, provided the quantity of growth factors in them corresponds to the developed standards to ensure efficacy of regenerative and reparative processes in the human body.

## References

[1] Brown S.A., Levi B., Lequex C., Wong V.W., Mojallal A., Longaker M.T (2010). Basic science review on adipose tissue for clinicians.. Plast Reconstr Surg.

[2] Turner A., Abu-Ghname A., Davis M.J., Winocour S.J., Hanson S.E., Chu C.K (2020). Fat grafting in breast reconstruction.. Semin Plast Surg.

[3] Rigotti G., Marchi A., Galiè M., Baroni G., Benati D., Krampera M., Pasini A., Sbarbati A. (2007). Clinical treatment of radiotherapy tissue demage by lipoaspirate transplant: a healing process mediated by adipose-derived adult stem cells.. Plast Reconstr Surg.

[4] Locke M.B., de Chalain T.M. (2008). Current practice in autologous fat transplantation: suggested clinical guidelines based on a review of recent literature.. Ann Plast Surg.

[5] Fernandes G., Yang S (2016). Application of platelet-rich plasma with stem cells in bone and periodontal tissue engineering.. Bone Res.

[6] Kon E., Filardo G., Di Matteo B., Marcacci M. (2013). PRP for the treatment of cartilage pathology.. Open Orthop J.

[7] Gonshor A (2002). Technique for producing platelet-rich plasma and platelet concentrate: background and process.. Int J Periodontics Restorative Dent.

[8] Marx R.E., Carlson E.R., Eichstaedt R.M., Schimmele S.R., Strauss J.E., Georgeff K.R (1998). Platelet-rich plasma: growth factor enhancement for bone grafts.. Oral Surg Oral Med Oral Pathol Oral Radiol Endod.

[9] Marx R.E (2001). Platelet-rich plasma (PRP): what is PRP and what is not PRP?. Implant Dent.

[10] Jain N.K., Gulati M (2016). Platelet-rich plasma: a healing virtuoso.. Blood Res.

[11] Marx R.E (2015). In situ tissue engineering bone regeneration in jaw reconstructiong.. Transl Regen Med.

[12] Kevy S.V., Jacobson V.S (2004). Comparison of methods for point of care preparation of autologous platelet gel.. J Extra Corpr Technol.

[13] Alves R., Grimalt R (2018). A review of platelet-rich plasma: history, biology, mechanism of action, and classification.. Skin Appendage Disord.

[14] Eppley B.L., Woodell J.E., Higgins J (2004). Platelet quantification and growth factor analysis from platelet-rich plasma: implications for wound healing.. Plast Reconstr Surg.

[15] Weibrich G., Kleis W.K., Kunz-Kostomanolakis M., Loos A.H., Wagner W (2001). Correlation of platelet concentration in platelet-rich plasma to the extraction method, age, sex, and platelet count of the donor.. Int J Oral Maxillofac Implants.

[16] Issa J.P.M., Tiossi R., da Silva Mello A.S., Lopes R.A., Di Matteo M.A.S., Iyomasa M.M. (2007). PRP: a possibility in regenerative therapy.. Int J Morphol.

[17] Sarban S., Tabur H., Baba F., Işıkan U.E. (2018). The positive impact of platelet-derived growth factor on the repair of full-thickness defects of articular cartilage.. Eklem Hastalik Cerrahisi.

[18] Samadi P., Sheykhhasan M., Khoshinani H.M (2019). The use of platelet-rich plasma in aesthetic and regenerative medicine: a comprehensive review.. Aesthetic Plast Surg.

[19] Masuki H., Okudera T., Watanebe T., Suzuki M., Nishiyama K., Okudera H., Nakata K., Uematsu K., Su C.Y., Kawase T (2016). Growth factor and pro-inflammatory cytokine contents in platelet-rich plasma (PRP), plasma rich in growth factors (PRGF), advanced platelet-rich fibrin (A-PRF), and concentrated growth factors (CGF).. Int J Implant Dent.

[20] Mazzocca A.D., McCarthy M.B.R., Chowaniec D.M., Cote M.P., Romeo A.A., Bradley J.P., Arciero R.A., Beitzel K (2012). Platelet-rich plasma differs according to preparation method and human variability.. J Bone Joint Surg Am.

[21] Ehrenfest D.M., Bielecki T., Mishra A., Borzini P., Inchingolo F., Sammartino G., Rasmusson L., Everts P.A (2012). In search of a consensus terminology in the field of platelet concentrates for surgical use: platelet-rich plasma (PRP), platelet-rich fibrin (PRF), fibrin gel polymerization and leukocytes.. Curr Pharm Biotechnol.

[22] Zorina A.I., Zorin V.L., Cherkasov V.R (2013). PRP in aesthetic medicine.. Eksperimental’naya i klinicheskaya dermatokosmetologiya.

[23] Mishra A., Pavelko T (2006). Treatment of chronic elbow tendinosis with buffered platelet-rich plasma.. Am J Sports Med.

[24] Wasterlain A.S., Braun H.J., Dragoo J.L (2012). Contents and formulations of platelet-rich plasma.. Oper Tech Orthop.

[25] Startseva O.I., Melnikov D.V., Zakharenko A.S., Kirillova K.A., Pishchikova E.D., Epifanova M.V., Safronov V.V., Istranov A.L. (2018). Method of augmentation mammoplasty..

[26] Andia I., Sánchez M., Maffulli N. (2012). Joint pathology and platelet-rich plasma therapies.. Expert Opin Biol Ther.

[27] Eppley B., Pietrzak W.S., Blanton M (2006). Platelet-rich plasma: a review of biology and applications in plastic surgery.. Plast Reconstr Surg.

[28] Kazakos K., Lyras D.N., Verettas D., Tilkeridis K., Tryfonidis M (2009). The use of autologous PRP gel as an aid in the management of acute trauma wounds.. Injury.

[29] Dohan D.M., Choukroun J., Diss A., Dohan S.L., Dohan A.J., Mouhyi J., Gogly B (2006). Platelet-rich fibrin (PRF): a second-generation platelet concentrate. Part II: platelet-related biologic features.. Oral Surg Oral Med Oral Pathol Oral Radiol Endod.

[30] Agrawal A.A (2017). Evolution, current status and advances in application of platelet concentrate in periodontics and implantology.. World J Clin Cases.

